# A Randomized Clinical Trial of Linagliptin vs. Standard of Care in Patients Hospitalized With Diabetes and COVID-19

**DOI:** 10.3389/fendo.2021.794382

**Published:** 2021-12-22

**Authors:** Ran Abuhasira, Irit Ayalon-Dangur, Neta Zaslavsky, Ronit Koren, Mally Keller, Dror Dicker, Alon Grossman

**Affiliations:** ^1^ Sackler Faculty of Medicine, Tel Aviv University, Tel Aviv, Israel; ^2^ Department of Internal Medicine B, Rabin Medical Center, Beilinson Campus, Petah Tikva, Israel; ^3^ Department of Internal Medicine E, Rabin Medical Center, Beilinson Campus, Petah Tikva, Israel; ^4^ Department of Internal Medicine A, Shamir (Assaf Harofeh) Medical Center, Zerifin, Israel; ^5^ Department of Internal Medicine D, Rabin Medical Center, Hasharon Campus, Petah Tikva, Israel

**Keywords:** COVID-19, diabetes, linagliptin, DPP-4 inhibitors, hospital management

## Abstract

**Objective:**

To assess the effect of linagliptin vs. standard therapy in improving clinical outcomes in patients hospitalized with diabetes and coronavirus disease 2019 (COVID-19).

**Materials and Methods:**

We did an open-label, prospective, multicenter, randomized clinical trial in 3 Israeli hospitals between October 1, 2020, and April 4, 2021. Eligible patients were adults with type 2 diabetes mellitus and a diagnosis of COVID-19. A total of 64 patients, 32 in each group, were randomized to receive linagliptin 5 mg PO daily throughout the hospitalization or standard of care therapy. The primary outcome was time to clinical improvement within 28 days after randomization, defined as a 2-point reduction on an ordinal scale ranging from 0 (discharged without disease) to 8 (death).

**Results:**

The mean age was 67 ± 14 years, and most patients were male (59.4%). Median time to clinical improvement was 7 days (interquartile range (IQR) 3.5-15) in the linagliptin group compared with 8 days (IQR 3.5–28) in the standard of care group (hazard ratio, 1.22; 95% CI, 0.70–2.15; p = 0.49). In-hospital mortality was 5 (15.6%) and 8 (25.0%) in the linagliptin and standard of care groups, respectively (odds ratio, 0.56; 95% CI, 0.16–1.93). The trial was prematurely terminated due to the control of the COVID-19 outbreak in Israel.

**Conclusions:**

In this randomized clinical trial of hospitalized adult patients with diabetes and COVID-19 who received linagliptin, there was no difference in the time to clinical improvement compared with the standard of care.

**Clinical Trial Registration:**

ClinicalTrials.gov, identifier NCT04371978.

## Introduction

The coronavirus disease 2019 (COVID-19) is an emerging pandemic in 2020–2021 caused by a novel coronavirus named severe acute respiratory syndrome coronavirus 2 (SARS-CoV-2) (1, 2). Diabetes confers a significant additional risk for COVID-19 patients (3–5). Currently, there are only a few specific effective therapeutic agents for the treatment of COVID-19 despite a large number of clinical trials that evaluated a wide range of therapies (6–10).

Dipeptidyl peptidase 4 (DPP-4) is a transmembrane glycoprotein expressed ubiquitously in many tissues and originally known as T-cell surface marker cluster of differentiation 26 (CD26) (11). In addition to its effect on glucose levels, DPP-4 has various effects on the immune system and several diseases, including lung diseases (11, 12). DPP-4 serves as the functional receptor for the Middle East respiratory syndrome coronavirus (MERS-CoV) (13), and potential interactions of SARS-CoV-2 spike glycoprotein and DPP-4 may play a significant role in the viral process of hijacking the human host cells (14).

In addition to their role in treating diabetes, several studies have evaluated the potential of DPP-4 inhibitors as immune-modulating agents and as a treatment for chronic allograft dysfunction following lung transplantation (11, 15). The anti-inflammatory effects of DPP-4 inhibitors and the possible involvement in the process of entering human cells are the basis for the assumption that these agents may be beneficial for the treatment of COVID-19 (16, 17). Two retrospective studies from Italy found that the use of DPP-4 inhibitors as a group or sitagliptin specifically reduced mortality and improved outcomes of patients hospitalized with COVID-19 (18, 19). Nevertheless, a meta-analysis based on retrospective studies concluded that the current data are insufficient and that the combined estimate of the risk ratio for mortality reduction by DPP-4 inhibitors is neutral (20).

Linagliptin is a DPP-4 inhibitor approved by the Food and Drug Administration in 2011 as a treatment for type 2 diabetes (21) and is as effective as other DPP-4 inhibitors (22). In contrast to other DPP-4 inhibitors, no dosage adjustment is necessary for renal or hepatic impairment, and linagliptin does not affect the risk of heart failure (23). In this multicenter randomized, controlled, open-label trial, we investigated the safety and efficacy of linagliptin in hospitalized patients with COVID-19 and diabetes.

## Materials and Methods

### Design

This was an investigator-initiated, multicenter, open-label randomized clinical trial aimed to assess the safety and efficacy of linagliptin vs. standard therapy in hospitalized patients with diabetes and COVID-19. A total of 3 hospitals in Israel enrolled patients between October 1, 2020, and April 4, 2021, from designated COVID-19 departments. Patients were randomized by the study coordinators using a web-based system with blocks of variable size to a 1:1 allocation ratio. Patients were randomized to receive linagliptin 5 mg daily PO plus standard of care or standard of care alone. The standard of care for patients with diabetes included holding oral drugs and initiating insulin therapy according to a basal bolus protocol (24). The goal of therapy was to keep blood glucose levels in the range of 140–180 mg/dl (25). COVID-19-specific treatments in both groups were according to the updated evidence (26). Treatment with linagliptin was continued from randomization to hospital discharge. The randomization was stratified by the hospital and in each hospital by age (≤70 or >70 years) and oxygen use at randomization (if supplemental oxygen was required or not). Patients were followed up until 28 days following randomization when they were contacted by the study staff by telephone. This trial followed the Consolidated Standards of Reporting Trials (CONSORT) reporting guidelines. Due to the control of the COVID-19 outbreak in Israel (27, 28), no patients were enrolled after April 4, 2021, and the trial was terminated prematurely on May 4, 2021.

### Patients

Hospitalized patients 18 years and older, with a diagnosis of COVID-19 confirmed by a positive reverse-transcriptase PCR (RT-PCR) assay for SARS-CoV-2 in a respiratory tract specimen, were all evaluated for eligibility for the study. Other inclusion criteria were a previous diagnosis of type 2 diabetes mellitus or a diagnosis initially made during hospitalization, according to the American Diabetes Association (ADA) guidelines (29), 10 days from COVID-19 symptom onset or within 48 h after positive RT-PCR test. Exclusion criteria included a need for mechanical ventilation or vasopressor medications prior to randomization; expected immediate need of intensive care unit (ICU) admission or immediate surgical intervention; current treatment with any DPP-4 inhibitor; pregnancy; or known hypersensitivity to DPP-4 inhibitors.

### Outcomes

The primary clinical endpoint was time to clinical improvement within 28 days after randomization. Clinical improvement was defined as a 2-point reduction in patients’ admission status on a 9-point ordinal scale. This scale has been used in different COVID-19 therapeutic trials (30–32) and was recommended by the R&D Blueprint of the WHO (33). The 9-point scale was as follows: 0, discharged, no clinical or virological evidence of infection; 1, discharged, no limitation of activities; 2, discharged, limitation of activities or ambulatory oxygen use; 3, hospitalized, no oxygen therapy; 4, hospitalized, oxygen by mask or nasal prongs; 5, hospitalized, non-invasive ventilation or high-flow oxygen; 6, hospitalized, intubation and mechanical ventilation; 7, hospitalized, ventilation + additional organ support—vasopressors, renal replacement therapy, extracorporeal membrane oxygenation; and 8, death.

Secondary outcomes were the proportion of patients with 2-point reduction in patients’ admission status on a 9-point ordinal scale; all-cause mortality at 28 days; in-hospital death; length of hospitalization; ICU admissions; mechanical ventilation; supplemental oxygen-free days at 28 days; ventilator-free days at 28 days; and the proportion of patients with 50% decrease in C-reactive protein (CRP) levels. Safety outcomes included treatment-related adverse events, serious adverse events, and premature discontinuations of the study drug.

### Statistical Analysis

During the initial design of the study and based on the trial of Cao et al. (30), assuming a decrease in the primary endpoint measure from a median of 16 days to a median of 8 days between the two trial groups with overall power of 80% and significance of 0.05, the sample size required for the trial was 88 patients. After publications of further trials, it was assumed that a larger sample size is required for such power, but the wide vaccine campaign and outbreak control in Israel (27, 28) led to premature termination of the trial.

Primary endpoint analysis was based on intention-to-treat analysis and included all the patients who had undergone randomization. The time to clinical improvement was assessed after all patients had reached day 28, with failure to reach clinical improvement or death before day 28 and before clinical improvement was considered as right-censored at day 28. The time to clinical improvement was portrayed by the Kaplan–Meier plot and compared with a log-rank test. Hazard ratios with 95% CIs were calculated by Cox proportional hazards model. Secondary outcomes such as mortality at 28 days, the proportion of ICU admission, the proportion of mechanical ventilation, and the proportion of CRP reduction were compared using the χ^2^ test. Only the p-value for the primary efficacy analysis (2-tailed; significance defined as p ≤ 0.05) was reported, and bilateral 95% CI was not adjusted for multiplicity for all the other comparisons. Statistical analyses were performed with Stata, version 13.0 (StataCorp, Texas, USA) and SPSS, version 25 (IBM Corp). This trial is registered with ClinicalTrials.gov, NCT04371978.

## Results

### Patients

**Figure 1:**
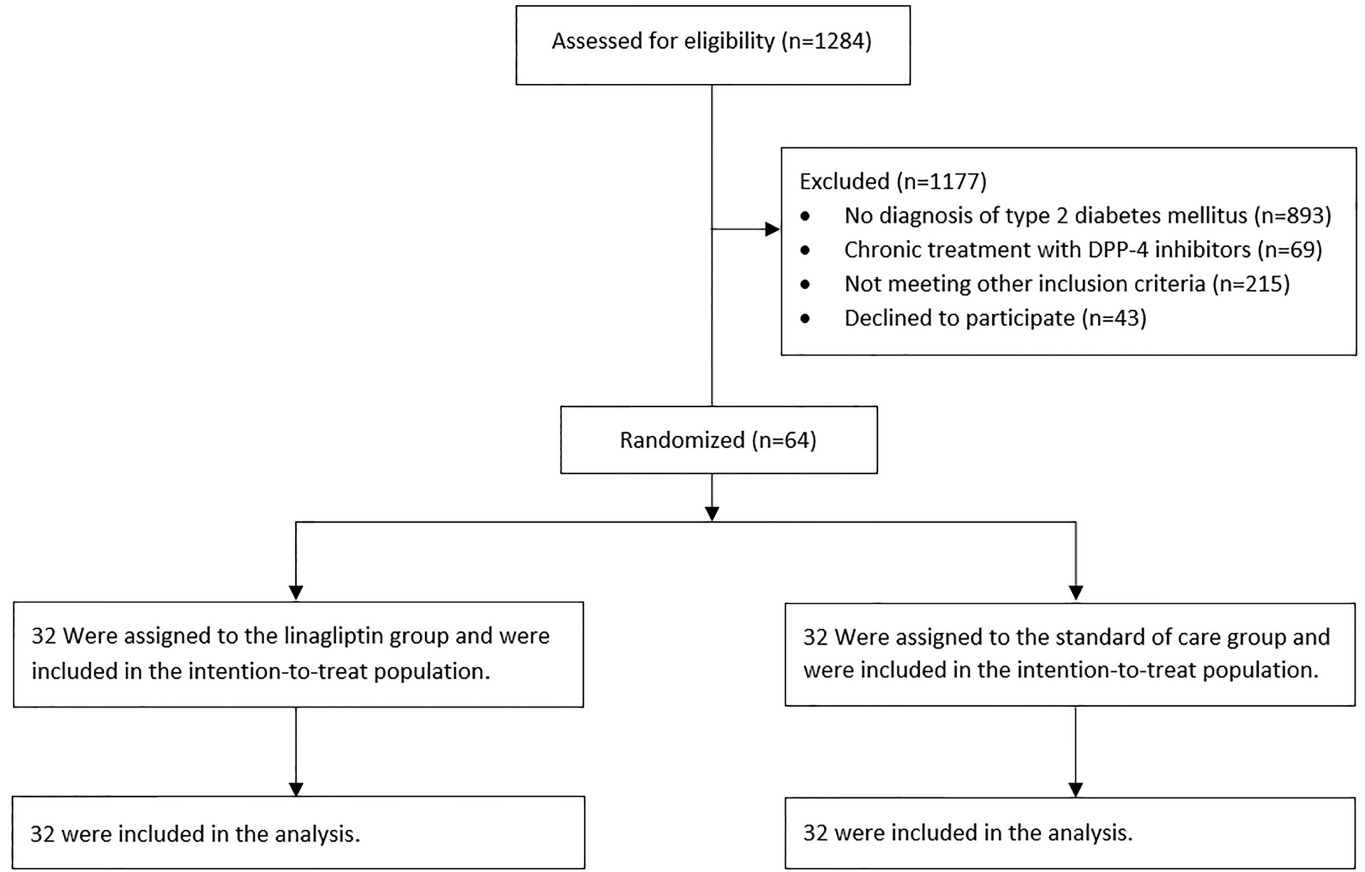
Flowchart of the study.

A total of 64 patients with COVID-19 from three hospitals in Israel underwent randomization and were included in this analysis. All patients were included in the intention-to-treat analysis (**Figure 1**). No patient withdrew from the trial. Demographic and baseline clinical characteristics are summarized in **Table 1**. Most of the patients were male (59.4%) with a mean age of 66.95 ± 13.93 years. Generally, the baseline clinical and laboratory parameters were balanced between the two groups, except for HbA1c, which was lower in the linagliptin group. COVID-19 disease classification was defined according to the National Institute of Health guidelines (10), and more than 70% of patients were classified as severe on admission. Of the patients, 84.4% and 81.3% received dexamethasone and 43.8% and 56.3% received remdesivir in the linagliptin and standard of care groups, respectively.

**Table 1:** Characteristics of the patients at baseline.

Characteristic	All patients (N = 64)	Linagliptin (n = 32)	Standard of care (n = 32)	p-Value
Age (years, mean ± SD)	66.95 ± 13.93	65.53 ± 16.02	68.38 ± 11.54	0.42
Male sex, n (%)	38 (59.38%)	21 (65.63%)	17 (53.13%)	0.31
Body mass index, median (IQR)	29.1 (26.6–33.3)	28.5 (26.3–33.3)	29.9 (26.7–33.3)	0.96
Oxygen support on admission, n (%)	41 (64.06%)	21 (65.63%)	20 (62.5%)	0.79
Duration of diabetes, years, median (IQR)	13.5 (8–19)	12.5 (5.5–20)	13.5 (8–18)	0.89
Diabetes complications, n (%)	28 (43.75%)	12 (37.5%)	16 (50%)	0.31
HbA1c median (IQR)				**0.02**
%	7.5 (6.7–8.8)	7.4 (6.6–7.9)	8.2 (6.8–9.3)	
mmol/mol	58 (50–73)	57 (49–63)	66 (51–78)	
Smokers, n (%)				0.11
Never	36 (56.25%)	16 (50%)	20 (62.5%)	
Former	23 (35.94%)	15 (46.88%)	8 (25%)	
Current	5 (7.81%)	1 (3.13%)	4 (12.5%)	
Charlson Comorbidity Index median (IQR)	7 (5–8.8)	6 (4–9)	7 (5–8)	0.69
Duration of symptoms prior to admission, days, median (IQR)	6 (2–8)	7 (2–8)	6 (3–8)	0.53
Disease severity on admission, n (%)				0.17
Mild	10 (15.63%)	3 (9.38%)	7 (21.88%)	
Moderate	8 (12.5%)	6 (18.75%)	2 (6.3%)	
Severe	46 (71.88%)	23 (71.88%)	23 (71.88%)	
**Chronic medications**				
Statins, n (%)	49 (76.56%)	24 (75%)	25 (78.13%)	0.77
Diuretics, n (%)	20 (31.25%)	8 (25%)	12 (37.5%)	0.28
Beta-blockers, n (%)	24 (37.5%)	11 (34.38%)	13 (40.63%)	0.61
Anti-platelets, n (%)	41 (64.06%)	18 (56.25%)	23 (71.88%)	0.19
Calcium channel blockers, n (%)	21 (32.81%)	11 (34.38%)	10 (31.25%)	0.79
ACEI/ARB, n (%)	39 (60.94%)	21 (65.63%)	18 (56.25%)	0.44
Steroids, n (%)	13 (20.31%)	7 (22.58%)	6 (18.75%)	0.71
**COVID-19 treatments**				
Remdesivir, n (%)	32 (50%)	14 (43.75%)	18 (56.25%)	0.32
Dexamethasone, n (%)	53 (82.81%)	27 (84.38%)	26 (81.25%)	0.74
Convalescent plasma, n (%)	7 (10.94%)	4 (12.5%)	3 (9.38%)	0.69
**Laboratory values on admission**				
Lymphocytes, absolute, median (IQR)	0.8 (0.6–1.2)	0.9 (0.6–1.3)	0.8 (0.5–1.2)	0.77
C-reactive protein, mg/dl, median (IQR)	9.6 (5.5–17.9)	9.9 (5.6–20.5)	8.5 (5–15.9)	0.32
LDH U/L, median (IQR)	682.5 (487.5–816.3)	687.5 (502.8–834)	633 (450–816.3)	0.74
Ferritin mg/ml, median (IQR)	439.4 (232.9–1,000.7)	574.2 (306.9–1,028.9)	363.2 (177–808.5)	0.64
D-dimer ng/ml, median (IQR)	1,024 (638.5–1,699)	926.5 (696.3–1,668.5)	1,114 (576–1,798)	0.27
Troponin ng/l, median (IQR)	20 (12–44)	12 (12–37.5)	23 (12–60)	0.33

ACEI, angiotensin-converting enzyme inhibitors; ARB, angiotensin receptor blockers; HbA1c, hemoglobin A1C; LDH, lactate dehydrogenase; IQR, interquartile range.

Bold p-values mean statistically significant p-value <0.05.

### Primary Outcome

**Figure 2:**
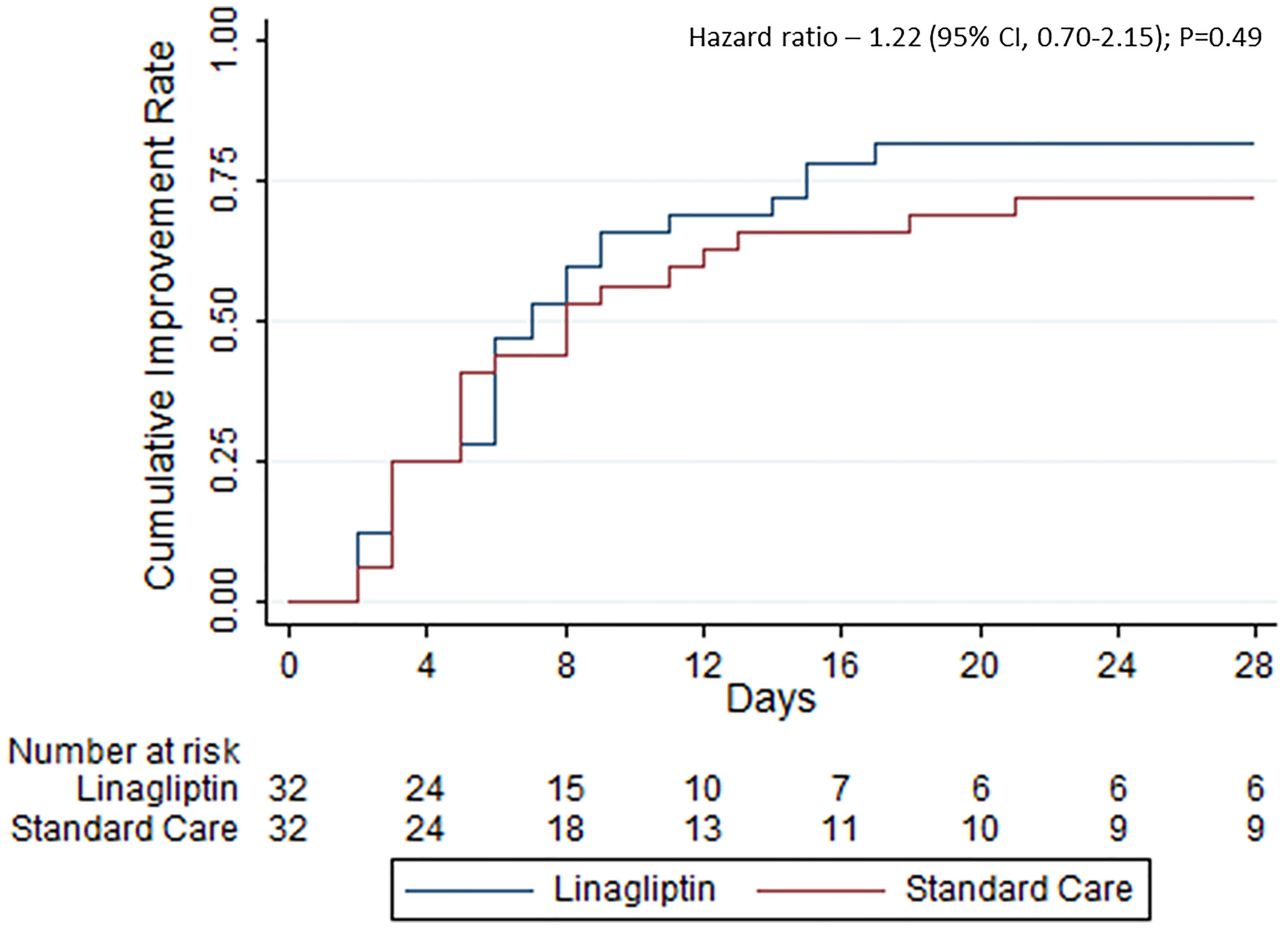
Kaplan–Meier estimates of cumulative clinical improvement.

A total of 26 patients (81.3%) in the linagliptin group and 23 (71.9%) in the standard of care group showed clinical improvement, i.e., 2-point reduction in the WHO scale, within 28 days of randomization (odds ratio, 0.59; 95% CI, 0.18–1.91) (**Table 2**). The time to improvement was also similar between the groups—median time of 7 days (interquartile range (IQR) 3.5–15) in the linagliptin group compared with 8 days (IQR 3.5–28) in the standard of care group (hazard ratio, 1.22; 95% CI, 0.70–2.15; p = 0.49, **Figure 2**). Different sensitivity analyses showed similar results.

**Table 2:** Outcomes in the intention-to-treat population.

Outcome	Linagliptin (n = 32)	Standard of care (n = 32)	Odds ratio (95% CI)	Mean difference (95% CI)	p-Value
Death at 28 days, n (%)	7 (21.9%)	9 (28.1%)	0.72 (0.23–2.23)		
In-hospital death, n (%)	5 (15.6%)	8 (25.0%)	0.56 (0.16–1.93)		
ICU admissions, n (%)	7 (21.9%)	4 (12.5%)	1.96 (0.51–7.50)		
Number of patients with 2-point reduction in WHO scale, n (%)	26 (81.25%)	23 (71.88%)	0.59 (0.18–1.91)		
Oxygen supplementation during the admission, n (%)	29 (90.6%)	25 (78.1%)	2.71 (0.63–11.59)		
Mechanical ventilation, n (%)	6 (18.8%)	4 (12.5%)	1.62 (0.41–6.38)		
CRP reduction of over 50% from admission to discharge, n (%)	14 (43.75%)	11 (34.38%)	0.67 (0.25–1.85)		
Length of hospitalization Mean ± SD Median (IQR)	8.81 ± 6.98 7 (3.5–11.5)	8.38 ± 6.086.5 (3–12.75)		0.44 (−2.83 to 3.71)	0.88
Supplemental oxygen-free days at 28 days Mean ± SD Median (IQR)	0.84 ± 1.71 0 (0–1)	1.44 ± 2.58 0 (0–2)		−0.59 (−1.69 to 0.5)	0.23
Ventilator-free days at 28 days Mean ± SD Median (IQR)	7.56 ± 5.90 6 (3–9)	7.25 ± 5.52 6 (3–11.25)		0.31 (−2.54 to 3.17)	0.77
ICU-free days at 28 days Mean ± SD Median (IQR)	7.75 ± 8.31 6 (3–9)	7.47 ± 5.29 6 (3–11.25)		0.28 (−3.2 to 3.76)	0.77

ICU, intensive care unit; CRP, C-reactive protein; IQR, interquartile range.

Adverse events related to the study drug were rare. Only two patients had a related adverse event, hypoglycemia, with one in the linagliptin group and the other in the standard of care group.

### Secondary Outcomes

There were no major differences in the rate of ICU admission between the groups, with 7 (21.9%) in the linagliptin group and 4 (12.5%) in the standard of care group (odds ratio, 1.96; 95% CI, 0.51–7.50) (**Table 2**). All-cause mortality at 28 days was 7 (21.9%) and 9 (28.1%) in the linagliptin and standard of care groups, respectively (odds ratio, 0.72; 95% CI, 0.23–2.23). The length of hospitalization was also similar in both groups with a median of 7 days (IQR 3–12) for the entire cohort (mean difference, 0.44; 95% CI, −2.83 to 3.71). One patient in the linagliptin group was still hospitalized in the ICU after 28 days from randomization.

## Discussion

Observational studies have suggested that DPP-4 inhibitors are effective in reducing mortality in patients with COVID-19 (18, 19). However, this randomized trial found that in hospitalized adults with COVID-19 and diabetes, linagliptin treatment added to the standard of care failed to significantly improve the time to resolution of symptoms or day 28 mortality. Furthermore, no difference between the study groups was observed for any of the secondary outcomes, including the proportion of patients admitted to an ICU, mechanical ventilation rates, length of hospitalization, and supplemental oxygen use. To our knowledge, this is the first randomized clinical trial evaluating the efficacy and safety of a DPP-4 inhibitor in patients with COVID-19.

As in many COVID-19 trials (31, 34–36), our study also had a male predominance in both groups, but the mean age was higher. The death and complications rates of our patients were higher than in most other clinical trials since our population included only patients with diabetes mellitus, a known risk factor for severe COVID-19 (3–5). The number of comorbidities was also high as reflected in the Charlson Comorbidity Index with a median score of 7, which is a strong predictor for mortality (37). As in other places in the world (38), hospital resources became scarce during the pandemic, and ambulatory treatment models were widely adopted. That narrowed hospitalizations only to those who needed them the most and explains why more than 70% of the patients were classified with severe disease on admission. Patients with a mild or moderate disease on admission were at high risk of progression to severe disease and/or with several additional acute illnesses.

The timing of linagliptin administration during the course of the disease might also be of importance. There are two suggested mechanisms for the effects of linagliptin on COVID-19. The first is the interaction of the virus with the human host cell (14), and the second is the anti-inflammatory effect (39). The first encourages use during the initial infection, while most patients are asymptomatic/presymptomatic, while the second is aimed for a later stage in the natural history of the disease, the inflammatory stage (40). Since we enrolled only hospitalized patients, treatment during the presymptomatic stage was not practical, but we enrolled patients only until the 10th day of symptoms. The results of previous observational studies support the hypothesis of the anti-inflammatory effect, as patients treated with DPP-4 inhibitors prior to COVID-19 infection had similar outcomes compared with those not being treated, as opposed to the studies in which DPP-4 inhibitors were used during the hospitalization, in which mortality reduction was shown (20). In our study, analysis of patients with symptoms shorter or longer than 5 days prior to admission showed no significant difference in the primary outcome. In addition, since most patients in our study were hospitalized with a disease classified as severe, it is possible that treatment of patients with diabetes and mild COVID-19 in the symptomatic stage may lead to different clinical outcomes. The use of DPP-4 inhibitors as anti-inflammatory agents in patients without diabetes may also lead to different clinical outcomes than those presented in this manuscript. Glucagon-like peptide 1 receptor (GLP-1R) agonists may have a similar anti-inflammatory effect in patients with diabetes and COVID-19, as they are known to reduce the levels of several anti-inflammatory markers (41). This should be assessed in further studies.

The Italian observational studies that showed mortality reduction with DPP-4 inhibitors were conducted during the early phase of the pandemic, February–April 2020 (18, 19). The rapid changes of clinical guidelines as new data emerged weekly led to a substantial difference between the treatment of COVID-19 patients in April 2020 and April 2021 (26, 42). Thus, it is possible that DPP-4 inhibitors have a beneficial effect on COVID-19 compared with supportive care alone, but this effect is eliminated when other therapies such as remdesivir and dexamethasone are added, as was for most of the patients in our study. The relation between the immunomodulatory effect of DPP-4 inhibitors and corticosteroids is unknown, and it is possible that dexamethasone diminished the immunomodulatory effect of linagliptin (43, 44). It should be noted that while sitagliptin specifically was evaluated in a previous study (18), we evaluated linagliptin, but if DPP-4 inhibitors are beneficial, then this is probably a class effect.

The very low rate of adverse events can be explained by the fairly good safety profile of linagliptin, especially with regard to severe adverse events (45). Another explanation is the short treatment period, with most patients treated for less than 10 days, while for diabetes, it is a chronic treatment, and most trials that evaluated the safety of the drug followed up patients for 6–24 months.

Our study has several limitations. The first limitation is the sample size and the premature discontinuation of the trial due to the end of the COVID-19 outbreak in Israel (27, 28). This makes our study underpowered to detect possible differences in the primary outcome and in mortality. The second limitation is the fact that the trial was open-label. We considered conducting a double-blind placebo-controlled trial, but that was not feasible due to the time constraints.

## Conclusions

The administration of linagliptin in patients with COVID-19 and diabetes did not improve the time to clinical improvement or 28-day mortality. Further large-scale, blinded, placebo-controlled randomized clinical trials are needed to confirm the results and to explore possible applications of DPP-4 inhibitors in different stages of the disease in patients with and without diabetes.

## Data Availability Statement

The data used in the analysis of this study are not publicly available due to the requirements of the IRB Committee but are available from the corresponding author upon request.

## Ethics Statement

The trial was approved by the institutional review board of Rabin Medical Center (confirmation number 0303-20-RMC) and Shamir Medical Center (confirmation number 0010-21-ASF). Written informed consent was obtained from all patients. The trial was conducted in accordance with the principles of the Declaration of Helsinki. The authors were responsible for designing the trial and for compiling and analyzing the data. The authors vouch for the completeness and accuracy of the data and for the adherence of the trial to the protocol. The participants provided their written informed consent to participate in this study.

## Author Contributions

Conceptualization: RA, AG, and IA-D. Methodology: RA and IA-D. Formal analysis: RA, NZ, MK, and DD. Investigation: RA and AG. Resources: AG. Data curation: RA. Writing—original draft preparation: all authors. Writing—review and editing: RA, AG, and RK. Supervision: AG and RK. Funding acquisition: AG. All authors have read and agreed to the published version of the manuscript.

## Funding

The study was funded by the internal budget of the Department of Internal Medicine B, Beilinson Campus, Rabin Medical Center.

## Conflict of Interest

The authors declare that the research was conducted in the absence of any commercial or financial relationships that could be construed as a potential conflict of interest.

## Publisher’s Note

All claims expressed in this article are solely those of the authors and do not necessarily represent those of their affiliated organizations, or those of the publisher, the editors and the reviewers. Any product that may be evaluated in this article, or claim that may be made by its manufacturer, is not guaranteed or endorsed by the publisher.
