# Supplementary material for: A Randomized Clinical Trial of Linagliptin vs. Standard of Care in Patients Hospitalized With Diabetes and COVID-19
Source: Front Endocrinol (Lausanne). 2021 Dec 22;12:794382. doi: 10.3389/fendo.2021.794382 (PMC8727772; doi:10.3389/fendo.2021.794382)
Supplement: Supplementary file 1 [file Table_1.docx]

Supplementary Material

**Supplemental Table 1 – Laboratory values of all patients on admission**

| **Characteristic** | **All patients (N=64)** | **Linagliptin (n=32)** | **Standard of care (n=32)** |
| --- | --- | --- | --- |
| Hemoglobin (g/dl, median, IQR) | 12.8 (11.3-14.3) | 13.1 (11.6-14.4) | 12.4 (10.8-14.2) |
| Platelets (K/micL, median, IQR) | 198.5 (157.8-239) | 204.5 (163.5-238.3) | 192.5 (139.8-254.8) |
| White blood cells (K/micL, median, IQR) | 5.6 (4.5-7.6) | 6.2 (4.9-8.6) | 5.2 (4.2-7.3) |
| Lymphocytes (absolute, median, IQR) | 0.8 (0.6-1.2) | 0.9 (0.6-1.3) | 0.8 (0.5-1.2) |
| Neutrophils (absolute, median, IQR) | 4.2 (3.2-5.7) | 4.5 (3.9-6.6) | 4.1 (3-5.4) |
| Creatinine (mg/dl, median, IQR) | 0.9 (0.8-1.3) | 0.9 (0.7-1.3) | 1 (0.8-1.5) |
| AST (U/L, median, IQR) | 29.5 (21.3-47) | 32.5 (23.5-50) | 28 (20.3-46.5) |
| ALT (U/L, median, IQR) | 21 (15-29) | 23.5 (15-43.2) | 18.5 (15-27.8) |
| GGT (U/L, median, IQR) | 51 (33-79) | 50 (33.5-104) | 55.5 (31-70.8) |
| ALP (U/L, median, IQR) | 68.5 (57.3-91) | 65.5 (51.5-84.5) | 70 (58.3-95.3) |
| Total Bilirubin (mg/dl, median, IQR) | 0.4 (0.3-0.6) | 0.4 (0.3-0.6) | 0.4 (0.3-0.6) |
| Albumin (g/dl, mean ± SD) | 3.59 ± 0.42 | 3.65 ± 0.41 | 3.53 ± 0.43 |
| C-reactive protein (mg/dl, median, IQR) | 9.6 (5.5-17.9) | 9.9 (5.6-20.5) | 8.5 (5-15.9) |
| LDH (U/L, median, IQR) | 682.5 (487.5-816.3) | 687.5 (502.8-834) | 633 (450-816.3) |
| Ferritin (mg/ml, median, IQR) | 439.4 (232.9-1000.7) | 574.2 (306.9-1028.9) | 363.2 (177-808.5) |
| Fibrinogen (mg/dl, mean ± SD) | 768.55 ± 185.49 | 772.29 ± 185.39 | 764.55 ± 188.78 |
| INR | 1.1 (1-1.2) | 1.1 (1-1.1) | 1.1 (1-1.2) |
| D-dimer (ng/ml, median, IQR) | 1024 (638.5-1699) | 926.5 (696.3-1668.5) | 1114 (576-1798) |
| Troponin (ng/l, median, IQR) | 20 (12-44) | 12 (12-37.5) | 23 (12-60) |

Abbreviations: AST - Aspartate aminotransferase; ALT - Alanine aminotransferase; GGT - Gamma glutamyl transferase; ALP - Alkaline phosphatase; LDH - Lactate dehydrogenase; INR - International normalized ratio.

**Supplemental Table 2 – Comorbidities of all patients**

| **Disease** | **All patients (N=64)** | **Linagliptin (n=32)** | **Standard of care (n=32)** |
| --- | --- | --- | --- |
| Chronic obstructive pulmonary disease | 6 (9.38%) | 4 (12.5%) | 2 (6.25%) |
| Asthma | 2 (3.13%) | 0 (0%) | 2 (6.25%) |
| Peripheral arterial disease | 9 (14.06%) | 2 (6.25%) | 7 (21.88%) |
| Myocardial infarction | 16 (25%) | 6 (18.75%) | 10 (31.25%) |
| Ischemic heart disease | 23 (35.94%) | 10 (31.25%) | 13 (40.63%) |
| Heart failure | 15 (23.44%) | 7 (21.88%) | 8 (25%) |
| Hypertension | 53 (82.81%) | 26 (81.25%) | 27 (84.38%) |
| CVA/TIA | 10 (15.63%) | 6 (18.75%) | 4 (12.5%) |
| Dementia | 3 (4.69%) | 2 (6.25%) | 1 (3.13%) |
| Cirrhosis | 4 (6.25%) | 3 (9.38%) | 1 (3.13%) |
| Peptic ulcer disease | 5 (7.81%) | 3 (9.38%) | 2 (6.25%) |
| Chronic kidney disease | 17 (26.56%) | 10 (31.25%) | 7 (21.88%) |
| Malignancy | 4 (6.25%) | 2 (6.25%) | 2 (6.25%) |
| Transplanted organs  Lung  Kidney | 8 (12.5%)  1 (1.56%)  5 (7.81%) | 6 (18.75%)  1 (3.13%)  4 (12.5%) | 2 (6.25%)  0 (0%)  1 (3.13%) |

Abbreviations: CVA/TIA – cerebrovascular accident/transient ischemic attack.

**Supplemental Table 3 – COVID-19 presenting symptoms**

| **Disease** | **All patients (N=64)** | **Linagliptin (n=32)** | **Standard of care (n=32)** |
| --- | --- | --- | --- |
| Fever or chills | 36 (56.25%) | 15 (46.88%) | 21 (65.63%) |
| Cough | 39 (60.94%) | 20 (62.5%) | 19 (59.38%) |
| Dyspnea | 48 (75%) | 25 (78.13%) | 23 (71.88%) |
| Loss of taste or smell | 6 (9.38%) | 4 (12.5%) | 2 (6.25%) |
| Weakness | 28 (43.75%) | 16 (50%) | 12 (37.5%) |
| Fatigue | 7 (10.94%) | 2 (6.25%) | 5 (15.63%) |
| Muscle ache | 8 (12.5%) | 4 (12.5%) | 4 (12.5%) |
| Headache | 4 (6.25%) | 3 (9.38%) | 1 (3.13%) |
| Sore throat | 3 (4.69%) | 1 (3.13%) | 2 (6.25%) |
| Congestion or runny nose | 1 (1.56%) | 1 (3.13%) | 0 (0%) |
| Diarrhea | 11 (17.19%) | 5 (15.63%) | 6 (18.75%) |
| Nausea or vomiting | 4 (6.25%) | 1 (3.13%) | 3 (9.38%) |
| Asymptomatic | 1 (1.56%) | 0 (0%) | 1 (3.13%) |
